# Two New Cyanogenic Glucosides from the Leaves of *Hydrangea macrophylla*

**DOI:** 10.3390/molecules17055396

**Published:** 2012-05-08

**Authors:** Chun-Juan Yang, Zhi-Bin Wang, Da-Ling Zhu, Ying Yu, Yin-Ting Lei, Yan Liu

**Affiliations:** 1 College of Pharmacy, Harbin Medical University, No. 157 Baojian Road, Nangang District, Harbin 150081, China; Email: chunjuanyang@126.com (C.-J.Y.); dalingz@yahoo.com (D.-L.Z.); 2 Key Laboratory of Chinese Materia Medica (Ministry of Education), Heilongjiang University of Chinese Medicine, Harbin 150040, China; Email: wzbmailbox@126.com (Z.-B.W.); 763775361@qq.com (Y.Y.); yintinglei@gmail.com (Y.-T.L.); 3 Department of Pharmacy, the Second Affiliated Hospital of Harbin Medical University, Harbin 150086, China

**Keywords:** Hydrangea *macrophylla*, Saxifragaceae, cyanogenic glucoside

## Abstract

Chemical investigation of the ethanol extract of the aerial parts of *Hydrangea macrophylla* collected in the Sichuan Province of China resulted in the isolation of two new cyanogenic glucosides. Their structures were elucidated as [(2*R*)-2-(*β*-D-glucopyranosyloxy)-2-(3,4-dimethoxy-phenyl)] acetonitrile (**1**) and {(2*R*)-2-[*α*-D-glucopyranosyl(1→6)*β*-D-glucopyranosyloxy]-2-(3-hydroxy-4-methoxy-phenyl)}acetonitrile (**2**) on the basis of extensive spectroscopic analysis (1D, 2D NMR and HRESIMS) and chemical studies.

## 1. Introduction

**Figure 1:**
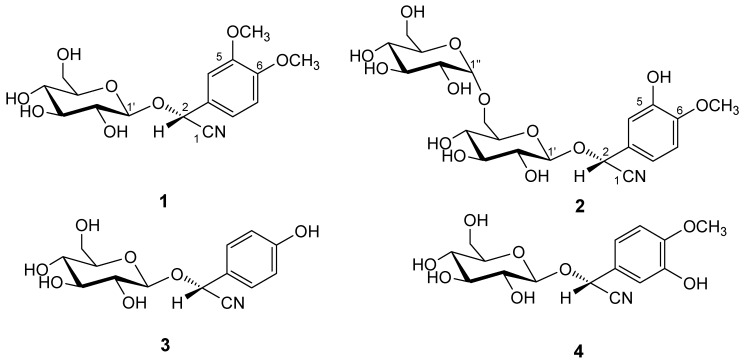
Structures of compounds **1**–**4**.

*Hydrangea macrophylla* (Thunb.) Ser., a Saxifragaceae plant, is widely cultivated in many countries, including China and Japan. Known biologically active components from the genus *Hydrangea* are dihydroisocoumarins and their glycosides [1,2,3], iridoid glycosides, secoiridoid glycosides [4,5,6], flavonoid glycoside [7] and cyanoglycosides. Pharmacology research has demonstrated that these compounds possessed many biological functions, such as antidiabetic [8], antiallergic, antimicrobial activities [2] and protective effect against rat liver injury induced by D-galactosamine [9]. In China, *H. macrophylla* is considered as a toxic Traditional Chinese Medicine, but there is no scientific evidence to illustrate the toxicity of this plant. According to the prior literature, researches concerning food poisoning cases in Japan were able to prove the presence of cyanogenic glycosides in *H. macrophylla* [10]. In addition, several novel non-cyanogenic cyanoglycosides were isolated from this plant, and these rare non-cyanogenic cyanoglycosides possessed some interesting bioactivities. For example, they were found to enhance the absorption of drugs, vitamins, and nutrients through the gastrointestinal membrane [11]. In the course of our chemical and pharmacological studies on *Hydrangea* plants, we tried to examine the chemical constituents from the aerial parts of *H. macrophylla* and isolated two new cyanogenic glycosides, [(2*R*)-2-(*β*-D-glucopyranosyloxy)-2-(3,4-dimethoxyphenyl)] acetonitrile (**1**) and {(2*R*)-2-[*α*-D-glucopyranosyl(1→6)*β*-D-glucopyranosyloxy]-2-(3-hydroxy-4-methoxyphenyl)}acetonitrile (**2**), together with two known cyanogenic glycosides, taxiphyllin (**3**) and hydracyanoside A (**4**). This paper deals with the structure elucidation of these two new compounds (Figure 1). 

## 2. Results and Discussion

**Figure 2:**
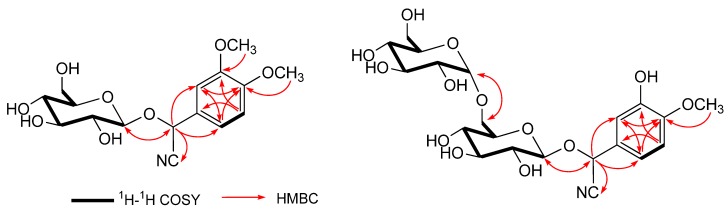
Key ^1^H-1H COSY and HMBC correlations of compounds **1** and **2**.

Compound **1** was obtained as a white amorphous powder. The IR spectrum of **1** showed an absorption band at 2,380 cm^−1^ ascribable to the cyano group. In the positive-ion ESIMS of **1**, a quasimolecular ion peak was observed at *m/z* 378 [*M*+Na]^+^. The molecular formula of **1** was found to be C_16_H_21_NO_8_ by HRFABMS analysis. Acid hydrolysis of **1** only liberated one D-glucose, which was identified by GC analysis using a hydrogen flame detector after treatment with L-cysteine methyl ester hydrochloride in pyridine [12]. The ^1^H-NMR (CD_3_OD) (Table 1) spectra of **1** indicated the presence of two methoxy groups [*δ* 3.86 (3H, s), *δ* 3.85 (3H, s)], a methine bearing an oxygen function [*δ* 5.84 (1H, s, H-2)], an ABX-type aromatic ring [*δ* 7.19 (1H, d, *J* = 2.0 Hz, H-4), 7.01 (1H, d, *J* = 8.3 Hz, H-7), 7.12 (1H, dd, *J* = 2.0, 8.2 Hz, H-8)], a *β*-D-glucopyranosyl moiety [*δ* 4.18 (1H, d, *J* = 7.6 Hz, H-1')]. ^13^C-NMR (CD_3_OD) (Table 1) spectra of **1** revealed the existence of a cyano group (*δ* 119.5, C-1), a glucosyl group at *δ* (C) 101.2, 74.7, 77.9, 71.6, 78.4, 62.9. As shown in Figure 2, the ^1^H-^1^H COSY experiment on **1** indicated the presence of partial structures written in bold lines, and in the HMBC experiment, long-range correlations were observed between the following protons and carbons: H-2 and C-1, 3, 4, 8; H-4 and C-2, 3, 5, 6, 8; H-atom of the methoxy group at *δ* (C) 56.6 and C-6. The site of attachment of sugar moiety on the aglycone of **1** as well as the position of the glucose linkage was also determined by HMBC experiment, which showed long-range correlations between the anomeric proton signal at *δ* 4.18 (H-1') and the carbon resonance at *δ* 68.0 (C-2). 

**Table 1:** ^1^H- and ^13^C-NMR data of **1** and **2** in CD_3_OD (*δ* in ppm, *J* in Hz, recorded at 600 MHz and 150 MHz, respectively).

Position	1		2		
	δ_C_	δ_H_		δ_C_	δ_H_
1	119.5			119.5	
2	68.0	5.84 (1H, s)		68.7	5.70 (1H, s)
3	127.0			127.1	
4	112.3	7.19 (1H, d, 2.0)		116.0	7.05 (1H, d, 2.0)
5	151.1			148.3	
6	152.0			150.6	
7	112.8	7.01 (1H, d, 8.3)		112.7	6.98 (1H, d, 8.9)
8	122.2	7.12 (1H, dd, 8.2, 2.0)		121.1	7.04 (1H, dd, 8.9, 2.0)
	56.5	3.85 (3H, s)		56.5	3.88 (3H, s)
	56.6	3.86 (3H, s)			
1'	101.2	4.18 (1H, d, 7.6)		101.8	4.26 (1H, d, 7.6)
2'	74.7	3.31 (1H, m)		74.7	3.28 (1H, m)
3'	77.9	3.24 (1H, m)		77.9	3.27 (1H, m)
4'	71.6	3.27 (1H, m)		71.9	3.30 (1H, m)
5'	78.4	3.20 (1H, m)		76.7	3.43 (1H, m)
6'	62.9	3.69 (1H, dd, 11.7, 2.1)		67.7	3.81 (1H, dd, 11.0, 2.0)
		3.92 (1H, dd, 11.7, 6.2)			3.92 (1H, dd, 11.0, 6.2)
1''				99.9	4.90 (1H, d, 3.0)
2''				73.7	3.42 (1H, t, 9.7,3.0)
3''				75.3	3.73 (1H, m)
4''				71.6	3.33 (1H, m)
5''				73.8	3.71 (1H, m)
6''				62.7	3.83 (1H, dd, 11.7, 2.1)
					3.70 (1H, dd, 11.7, 5.5)

**Figure 3:**
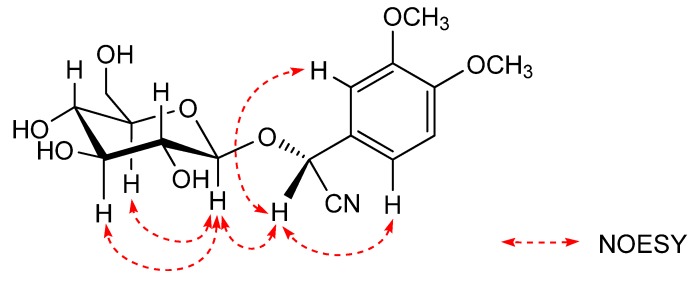
Key NOESY correlations of compound **1**.

The identification of the absolute stereostructure of compound **1** is based on the comparison of the chemical shifts of known cyanogenic glycosides in the ^1^H-NNR (CD_3_OD) and the NOESY experiment. According to the previous literature [10,13], the absolute configuration at the C-2 of **1** was determined by comparison of the 2- and 1'-proton signals of **1** with those of known cyanogenic glycosides. Namely, the 2- and 1'-proton signals in the ^1^H-NNR (CD_3_OD) spectrum of prunasin [*δ* 5.89 (H-2), 4.25 (H-1')] and taxiphyllin [*δ* 5.78 (H-2), 4.17 (H-1')] having *R* orientation were shifted upfield relative to those of sambunigrin [*δ* 6.03 (H-2), 4.67 (H-1')] and dhurrin [*δ* 5.90 (H-2), 4.67 (H-1')] having *S* orientation. The 2- and 1'-proton signals of **1** were observed at *δ* 5.84 and 4.18, respectively, so the C-2 orientation of **1** was assumed to be *R*. Also, the configuration at the 2-position in **1** was confirmed to be *R* by the NOESY experiment. Seigler *et al*. reported that a correlation was observed between the aromatic proton and the sugar core in the NOESY spectrum of the analogue of *S* orientation at C-2, and *R* orientation at C-2 was based on the observation of NOE interactions between the following protons:H-2 and H-1'(the sugar core); H-2 and the aromatic proton [14]. The NOE interactions of **1** were observed as shown in the Figure 3, C-2 orientation of **1** was determined to be *R* by comparison of the NOE interactions. Finally, **1** was determined as [(2*R*)-2-(*β*-D-glucopyranosyloxy)-2-(3,4-dimethoxy-phenyl)] acetonitrile.

Compound **2** was isolated as a white powder. The IR spectrum of **2** revealed the presence of the hydroxyl group (3,631 cm^−^^1^), cyano group (2,361 cm^−^^1^) and ether group (1,078 cm^−^^1^). The positive-ion FAB-MS of **2** showed a quasimolecular ion peak, which was observed at *m/z* 378 [*M*+Na]^+^. The molecular formula of **2** was found to be C_21_H_29_NO_13_ by HRFABMS analysis. Acid hydrolysis of **2** only gave D-glucose. The proton and carbon signals of **2** in the ^1^H and ^13^C-NMR spectra were almost superimposable with those reported for **1**, except for the signals due to 5'-position of the methoxy group and the 6'-position of the D-glucopyranosyl moiety on **2**. The planar structure of **2** was unambiguously confirmed by means of ^1^H-^1^H COSY and HMBC experiments (Figure 2), which showed long-range correlations between the following protons and carbons: H-2 and C-1', C-1, C-3, C-4, C-8; H-1' and C-2; H-1'' and C-6'. The signal of an anomeric proton at *δ* 4.90 (d, *J* = 3.0, H-1'') and the carbon at *δ* 99.9 suggested the presence of a sugar moiety in the *α*-form [15,16]. Based on the above-mentioned evidence, **2** was confirmed to be a cyanogenic diglycoside. Finally, by comparison of the 2- and 1-proton signals of **2** with those of known cyanogenic glycosides in the ^1^H-NNR (CD_3_OD) spectrum as that used to characterize the configuration at the C-2 position of **1**, the C-2 orientation of **2** was established to be R. Consequently, the structure of **2** was elucidated as {(2*R*)-2-[*α*-D-glucopyranosyl (1→6) *β*-D-glucopyranosyloxy]-2-(3-hydroxy-4-methoxy-phenyl)} acetonitrile.

The total amount of the four cyanogenic glycosides is more than 0.1% (3 kg material, compound **1** 77.5 mg, **2** 25.5 mg, **3** 5.5mg and **4** 2.91 g, respectively.) in the aerial parts of *H. macrophylla*. Nakamura *et al*. have reported that the amount of hydracyanoside A in the fresh leaves of *H. macrophylla* is 0.089% [10]. Under the action of the enzyme or acid conditions, the cyanogenic glucosides are able to decompose into HCN, and there are the cases of HCN poisoning if people eat excessive cyanogenic glycosides. The discovery of cyanogenic glucosides in *H. macrophylla* provides an explanation for the reported cases of food poisoning that present symptoms of vomiting, *etc*. after eating the leaves of *H*. *macrophylla*. Sendker and Nahrstedt reported that the cyanogenic glucosides could generate primary amide glucosides upon drying and decaying of the leaves [17], but we did not find any primary amide glucoside during the separation process. 

Many plants have been found to be rich in cyanogenic glycosides. The six cyanogenic glycosides [10] isolated from *H. macrophylla* were characterized to be *R* configuration at the C-2 position and have an ABX-type trisubstituted aromatic ring. And the distribution of cyanogenic glycosides with a trisubstituted aromatic ring in natural sources is very limited. It seems probable that nitrile glucosides with ABX-type benzene ring structures derive from tyrosine or phenylalanine [13].

## 3. Experimental

### 3.1. General

Open column chromatography was carried out using silica gel (200–300 mesh, Qingdao Marine Chemical Co., Qingdao, China) or octadecyl silica gel (ODS, 25–40 μm, Fuji, Tokyo, Japan) as stationary phase. TLC employed precoated silica gel plates (5–7 μm, Qingdao Marine, Qingdao, China). Preparative HPLC was carried out on a Waters 600 instrument equipped with a Waters UV-2487 detector. A Waters Sunfire prep C18 OBD (19 × 250 mm i.d.; Milford, PA, USA) column was used for preparative purpose. The IR spectra were recorded as KBr pellets on a Jasco 302-A spectrometer. Optical rotation was recorded on a Jasco P-2000 polarimeter. HRESIMS were measured on a FTMS-7 instrument (Bruker Daltonics, Karlsruhe, Germany). The ^1^H, ^13^C and 2D (^1^H-^1^H COSY, HSQC, HMBC, NOESY) NMR spectra were recorded on a JNM-ECA600 spectrometer using standard pulse sequence. Chemical shifts were reported in ppm (*δ*), and scalar coupling were reported in Hz. GC analyses were carried out using a Fuli 9790 instrument, DM-5 column (0.25 μm, 30 m × 0.25 mm, Dikma, Beijing, China). 

### 3.2. Plant

The aerial parts of *H. macrophylla* were collected in October 2008 from Shizhu of Sichuan Province, China and identified by Prof. Zhenyue Wang, of Heilongjiang University of Chinese Medicine. The voucher specimen (20081009) was deposited at herbarium of Harbin Medical University, Harbin, China.

### 3.3. Extraction and Isolation

The dried aerial parts of *H. macrophylla* (3.0 kg) were powdered and extracted 3 times with 70% EtOH (24 L) under reflux for 3 h. Evaporation of the solvent under reduced pressure provided a 70% EtOH extract (420.0 g), and the extract was partitioned into an EtOAc-H_2_O (1:1, *v*/*v*) mixture to furnish an EtOAc-soluble fraction (102.5 g) and an aqueous phase. The aqueous phase was further extracted with *n*-BuOH to give an *n*-BuOH-soluble fraction (154.5 g, 12.4%) and an H_2_O-soluble fraction (161.0 g). The *n*-BuOH-soluble fraction was subjected to ordinary-phase silica gel column chromatography [3.0 kg, CHCl3-MeOH-H2O (10:1; 5:1; 3:1; 2:1; 1:1, v/v)] to give seven fractions (1–7). 

Fraction 4 (10.7 g) was subjected to reversed-phase silica gel column chromatography (1:9; 2:8; 3:7; 4:6; 5: 5; 6: 4, *v*/*v*) to afford six fractions [Fr. 4-1 (336 mg), Fr. 4-2 (1.3 g), Fr. 4-3 (4.1 g), Fr. 4-4 (342 mg), Fr. 4-5 (412 mg), Fr. 4-6 (238 mg)]. Fr. 4-2 was separated by preparative HPLC using MeOH-H_2_O (1:9, *v*/*v*) into **1** (77.5 mg, *t*_R_ = 32.2 min). Fr. 4-3 was separated by preparative HPLC using MeOH-H_2_O (15:85, *v*/*v*) into **4** (2.91 g, *t*_R_ = 16.7 min). Fraction 5 (9.2 g) was subjected to reversed-phase silica gel column chromatography (1:9; 2:8; 3:7; 4:6; 5: 5; 6: 4, *v*/*v*) to afford five fractions [Fr. 5-1 (20.8 mg), Fr.5-2 (279 mg), Fr. 5-3 (432 mg), Fr. 5-4 (677 mg), Fr. 5-5(217 mg)]. Fr. 5-2 was separated by preparative HPLC using MeOH-H_2_O (12:88, v/v) into **2** (25.5 mg, *t*_R_ = 22.2 min). Fr. 5-3 was separated by preparative HPLC using MeCN-H_2_O (9:91, v/v) into **3** (5.5 mg, *t*_R_ = 22.2 min). The known compounds taxiphyllin [11] and hydracyanoside A [9] were identified by comparison of their spectral data ([*α*]_D_, ^1^H-NMR, ^13^C-NMR, MS) with reported literature values.

*[(2R)-2-( β- D-glucopyranosyloxy)-2-(3,4-dimethoxy -phenyl)] acetonitrile* (**1**): White amorphous powder. [*α*]25D –67.4°(*c* = 0.35, MeOH). UV (MeOH) *λ* max(log *ε* )nm: 237 (3.62), 282 (3.94). IR (KBr): *ν* = 3631, 2380, 1638, 1597, 1466, 1264, 1238, 1143, 1024, 856, 816, 766 cm^−^^1^. HRFABMS (positive): *m/z* 378.1159 (calc. for C_16_H_2__1_NO_8_, 378.1165, [*M*+Na]^+^), ^1^H and ^13^C-NMR (CD_3_OD) data: see Table 1.

*{(2R)-2 - [ α - D -glucopyranosyl(1 ° 6) β - D -glucopyrano syloxy] -2-(3-hydroxy-4-methoxy - phenyl)}* *acetonitrile* (**2**): White amorphous powder. [*α*] 25D–38.0°(*c* = 0.20, MeOH). UV (MeOH) *λ* max (log *ε*) nm: 280 (3.76), 238 (4.16). IR (KBr): *ν* = 3631, 2924, 2361, 1637, 1597, 1516, 1443, 1275, 1078, 871, 763 cm^−^^1^. ESIMS (positive): *m/z* 502.1582 (calc. for C_21_H_29_NO_13_, 503.1639, [*M*+Na]^+^), ^1^H and ^13^C-NMR (CD_3_OD) data: see Table 1.

### 3.4. Acid Hydrolysis of and Determination of the Absolute Configuration of the Monosaccharides

Compounds **1**, **2** (each 3.0 mg) was hydrolyzed with 1 M HCl (1.0 mL) for 2 h at 85 °C. The reaction mixture was cooled and partitioned between CHCl_3_ (2.0 mL) and H_2_O (2.0 mL). The aqueous layer was washed with CHCl_3_ (3.0 mL × 3), neutralized, filtered, and evaporated under reduced pressure. The residue was dissolved in pyridine (1.0 mL) and 0.1 M L-cysteine methyl ester hydrochloride in pyridine (2.0 mL) was added. The mixture was heated at 60 °C for 1 h. An equal volume of Ac_2_O was added with heating continued 1 h. The acetylated thiazolidine derivatives were analyzed by GC using a DM-5 Column (30 m × 0.25 mm, 0.25 μm). Temperatures of injector and detector were 280 °C for both. A temperature gradient system was used for the oven; starting at 160 °C and increasing up to 195 °C at a rate of 5 °C/min. Peaks of the hydrolysate were detected by comparison with retention time of authentic samples of D-glucose (10.08 min) after treatment with L-cysteine methyl ester hydrochloride in pyridine.

## 4. Conclusions

In this paper, two new cyanogenic glucosides, [(2*R*)-2-(*β*-D-glucopyranosyloxy)-2-(3, 4-dimethoxy-phenyl)] acetonitrile (**1**) and {(2*R*)-2-[*α*-D-glucopyranosyl(1→6)*β*-D-glucopyranosyloxy]-2-(3-hydroxy-4-methoxy-phenyl)}acetonitrile (**2**), were isolated together with two known cyanides from the EtOH extract of the dried aerial parts of *H. macrophylla*. The discovery of cyanogenic glucosides in the *H. macrophylla* provides the proof that the *H. macrophylla* is a kind of toxic TCM. The cyanogenic glucosides in *H. macrophylla* should be assayed in order to ensure safety of patients during treatment.

## References

[1] Hashimoto T., Tori M., Asakawa Y. (1987). Three dihydroisocoumarin glucosides from *Hydrangea macrophylla* subsp. serrata. Phytochemistry.

[2] Yoshikawa M., Uchida E., Chatani N., Kobayashi H., Naitoh Y., Okuno Y., Matsuda H., Yamahara J., Murakami N. (1992). Thunberginols C, D, and E, new antiallergic and antimicrobial dihydroisocoumarins, and thunberginol G 3'-*O*-glucoside and (−)-hydrangenol 4'-*O*-glucoside, new dihydroisocoumarin glycosides, from *Hydrangeae Dulcis* Folium. Chem. Pharm. Bull..

[3] Yoshikawa M., Ueda T., Shimoda H., Murakami T., Yamahara J., Matsuda H. (1999). Dihydroisocoumarin constituents from the leaves of *Hydrangea macrophylla* var*.* thunbergii (2). Absolute stereostructures of hydrangenol, thunberginol I, and phyllodulcin glycosides and isomerization reaction at the 3-positions of phyllodulcin and its glycosides. Chem. Pharm. Bull..

[4] Inouye H., Takeda Y., Uesato S., Uobe K., Hashimoto T., Shingu T. (1980). A novel type secoiridoid glucoside, hydrangenoside A from *Hydrangea macrophylla*. Tetrahedron Lett..

[5] Uesato S., Hashimoto T., Takeda Y., Uobe K., Inouye H. (1981). Novel type secoiridoid glucosides, hydrangenosides B, C and D from *Hydrangea macrophylla*. Chem. Pharm. Bull..

[6] Uesato S., Takeda Y., Hashimoto T., Uobe K., Inouye H., Taguchi H., Endo T. (1984). Studies on monoterpene glucosides and related natural products-absolute structures of Hydrangenosides A, B, C, D, E, F, and G. Novel type secoiridoid glucosides from two *Hydrangea* Plants. Helv. Chim. Acta.

[7] Murakami N., Mostaqul H.M., Tamura S., Itagaki S., Horii T., Kobayashi M. (2001). New anti-malarial flavonol glycoside from *Hydrangeae dulcis* Folium. Bioorg. Med. Chem. Lett..

[8] Zhang H.L., Hisashi M., Akria K., Yuki I., Seikou N., Masayuki Y. (2007). New type of anti-diabetic compounds from the processed leaves of *Hydrangeae macrophylla* seringe var*.* thunbergii (*Hydrangeae dulcis* Folium.). Bioorg. Med. Chem. Lett..

[9] Ryusuke N., Erika H., Shun K., Yasushi S., Toshikazu K. (2003). Suppresion by *Hydrangeae dulcis* Folium. of D-galactosamine-induced liver injury *in vitro* and *in vivo*. Biosci. Biotechnol. Biochem..

[10] Seikou N., Wang Z.B., Xu F.M., Hisashi M., Wu L.J., Masayuki Y. (2009). The absolute stereostructures of cyanogenic glycosides, hydracyanosides A, B, and C, from the leaves and stems of *Hydrangea macrophylla*. Tetrahedron Lett..

[11] Wang Z.B., Gao H.Y., Yang C.J., Sun Z., Wu L.J. (2011). Novel cyanoglucosides from the leaves of *Hydrangea macrophylla*. Helv. Chim. Acta.

[12] Yang C.J., An Q., Xiong Z.L., Song Y., Yu K., Li F.M. (2009). Triterpenes from *Acanthopanax sessiliflorus* fruits and their antiplatelet aggregation activities. Planta Med..

[13] Seigler D.S., Pauli G.F., Frohlich R., Wegelius E., Nahrstedt A., Glander K.E., Ebinger J.E. (2005). Cyanogenic glycosides and menisdaurin from *Guazuma ulmifolia*, *Ostrya virginiana*, *Tiquilia plicata* and *Tiquilia canescens*. Phytochemistry.

[14] Seigler D.S., Pauli G.F., Nahrstedt A., Leen R. (2002). Cyanogenic allosides and glucosides from *Passiflora edulis* and *Carica papaya*. Phytochemistry.

[15] Gorin P.A.J., Mazurek M. (1975). Further studies on the assignment of signals in ^13^C magnetic resonance spectra of aldoses and derived methyl glycosides. Can. J. Chem..

[16] Derek H., Jon S.J., Kerstin D.P. (1966). Anomeric equilibria in derivatives of amino sugars. Some 2-amino-2-deoxy-D-hexose derivatives. J. Org. Chem..

[17] Sendker J., Nahrstedt A. (2009). Generation of primary amide glucosides from cyanogenic glucosides. Phytochemistry.

